# Supplementary material for: The role of sex and handedness in the performance of the smartphone-based Finger-Tapping Test
Source: PLOS Digit Health. 2023 Aug 16;2(8):e0000304. doi: 10.1371/journal.pdig.0000304 (PMC10431671; doi:10.1371/journal.pdig.0000304)
Supplement: Attachment — Submitted filename: Brito et al Reply 220427.docx [file pdig.0000304.s002.docx]

Journal Requirements:

1. Please amend your detailed Financial Disclosure statement. This is published with the article. It must therefore be completed in full sentences and contain the exact wording you wish to be published.

a. State the initials, alongside each funding source, of each author to receive each grant.

b. If any authors received a salary from any of your funders, please state which authors and which funders.

A. Done.

2. Figures 1, 2, 4, and 6 contains screenshots. We are not permitted to publish these under our CC-BY 4.0 license; websites are usually intellectual property and are copyrighted. This includes peripheral graphics of the web browser such as the buttons. We ask that you please remove or replace it.

A. Dear Editor, we only used authorial images in our Figures built in Corel Draw, MATLAB and R Studio. Please, indicate which part is inadequate and we would be pleased to change.

Additional Editor Comments (if provided):

The topic of the manuscript is interesting. I appreciate if the authors address the following issues in their revision.

1-	For keywords, please consider using appropriate MeSH terms.

A. Done.

2-	In the introduction section, please add more information about the Finger-Tapping Test (FTT).

A. Done.

3-	In the introduction section, please expand justification for conducting the research.

A. Done.

4-	In the methods section, please provide the ethics approval code.

A. Done.

5-	In the methods section, please explain why only right-handed participants were included? What about the left-handed people?

A. Thanks for the comment. We added an explanation in the Methods section for the exclusion of left-handed participants.

6-	How did the researchers recruit the participants? Was there any facilitators to guide the participants during completing their tasks?

A. The participants in this study were recruited through convenience sampling from a university population and prior to the experiments, all participants underwent a training test and received instructions by the same experimenter to ensure they were familiar with the procedures and tasks. This step was taken to minimize any potential confusion or misunderstandings during the actual experiment and to ensure that all participants had a consistent level of understanding and preparation. We added this information in the Methods section.

7-	In the methods section, please separate the information for each protocol using appropriate subheadings.

A. Done

8-	Please follow the journal instructions for organizing the list of the references. Moreover, references older than 10 years ago need to be updated.

A. Done

REVIEWER #1

Overall, this is a clearly written and interesting manuscript that advances scientific knowledge in this field. I recommend publication but have some suggested edits to improve the manuscript.

General comments:

1. There are several instances of the incorrect abbreviation 'FFT' being used instead of 'FTT' - please correct this.

A. Done.

2. There is a lot of data presented in the Results section - the Forest plots are helpful but it is difficult to visualise the significance of the effects (particularly in the black and white figures sent). Perhaps statistical significance could also be incorporated into the tables - for example, highlighting in bold or using an asterisk system to indicate where the significant differences are?

A. We added the Effect Size information on the Tables. Thanks for the suggestions.

3. The figure legends and descriptions in the text do not appear to match the figure labels in Figures 2, 4 and 6 (e.g. "Figure 2E shows an example of the spatial distribution of the taps for a representative participant" - it doesn't, it shows the female dominant hand tapping frequency)

A. Sorry, It is a mistake. Thank you very much for the comment. We corrected the figure legends.

Specific comments:

INTRODUCTION

4. Paragraph 2 - please correct grammar, e.g. "Initially, it was very common TO use THE telegraph key..." and, "... image-based motion [7], AND musical instrument midi keyboard [8]".

A. We rewrote the sentence following the suggestion.

5. Paragraph 3 - please add further detail on the studies in references 15-22 so the reader does not have to search through the individual references. For example, what conditions were studied, in what contexts? More detail could also be added on the potential applications of the Finger-Tapping Test here and in the Discussion.

A. Done.

METHODS

6. Final paragraph - please correct grammar, e.g. "Figure 7 INDICATES that handedness..."

A. Done

DISCUSSION

7. Paragraph 1 - 'Adult people' is an odd term - please change to 'adults'.

A. Done.

8. Paragraph 2 - define handedness here, i.e. use of the dominant hand (so this does not get confused with left- and right-handedness). This might also need to be clarified in other sections as I first interpreted handedness to mean left- vs. right-handedness.

A. Thanks for the comment. We added in the Introduction section the concept of handedness.

9. Paragraph 2 - point (ii) would be clearer written as, "median frequency was significantly influenced by an interaction between sex and handedness..."

A. Done.

10. More detail could be added on the potential applications of the Finger-Tapping Test. Parkinson's Disease is mentioned frequently but are there other relevant conditions/clinical uses of the test? How does the FTT using a smartphone compare (in terms of accuracy and reliability) with FTTs using other technologies? Are there any limitations specific to smartphones? If there have not been studies on this, then this is an obvious area for future research.

A. Thanks. We added a paragraph in the Discussion section about the suggestion.

REVIEWER #2

Dear Editors,

Att: THE ROLE OF SEX AND HANDEDNESS IN THE PERFORMANCE OF THE SMARTPHONE-BASED FINGER-TAPPING TEST.

The proposed manuscript is a summative report of the role of sex and handedness in the performance of the smartphone–based FTT. In my opinion, the paper lacks clarity in writing due to specific reasons:

1. Upfront, the authors should state that an abbreviated version was used in the study. Please say why you chose an abbreviated version. What was the rationale for selecting the referenced abbreviated version? Do not let the reference do the work for you. Motivate your reasons for the tools/ instrument for this study.

A. Thanks for the comments. We added in the Method section that we chose an abbreviated version to short the testing duration.

2. The significance should be indicated earlier in the paper. The rationale for the novelty or significance of this test needs to be stated in the article.

A. Done.

3. Indicate the test’s administration time and include the breaks between the tests for each protocol. It is not evident from the design the duration of the test.

A. Done.

4. Remember to indicate whether this was a patient self–report on handedness.

A. Done

The authors need to tend to the writing of the abstract and the critical measurements not foregrounded at the beginning of the paper or in the method section. It needs to be apparent from findings the clinical and statistical significance of the role of sex and handedness. Without justification or rationale for the measurement, the authors lose the reader on why sex and handedness differ in practice for this clinical group of patients as opposed to the theoretical assumption of the validity of measuring sex for the adapted version of the FTT. You cannot only state that it is significant for future reference.

I propose that the authors address the lack of clarity in writing of the above-referenced feedback. Therefore, minor revision is required to consider for publication.

A. Thank you very much for all the suggestions.

--------------------
