# Supplementary material for: The role of sex and handedness in the performance of the smartphone-based Finger-Tapping Test
Source: PLOS Digit Health. 2023 Aug 16;2(8):e0000304. doi: 10.1371/journal.pdig.0000304 (PMC10431671; doi:10.1371/journal.pdig.0000304)
Supplement: Attachment — Submitted filename: Reply v2.docx [file pdig.0000304.s003.docx]

Journal Requirements:

1. Please review your reference list to ensure that it is complete and correct. If you have cited papers that have been retracted, please include the rationale for doing so in the manuscript text, or remove these references and replace them with relevant current references. Any changes to the reference list should be mentioned in the rebuttal letter that accompanies your revised manuscript. If you need to cite a retracted article, indicate the article's retracted status in the References list and also include a citation and full reference for the retraction notice.

A. Our reference list was not changed.

2. We have noticed that you have uploaded Supporting Information files, but you have not included a list of legends. Please add a full list of legends for your Supporting Information files after the references list.

A. Done.

3. Please ensure that Funding Information and Financial Disclosure Statement are matched.

A. Done.

Reviewers' comments:

Reviewer's Responses to Questions

1. If the authors have adequately addressed your comments raised in a previous round of review and you feel that this manuscript is now acceptable for publication, you may indicate that here to bypass the “Comments to the Author” section, enter your conflict of interest statement in the “Confidential to Editor” section, and submit your "Accept" recommendation.

Reviewer #1: (No Response)

Reviewer #2: All comments have been addressed

A. Ok

--------------------

2. Does this manuscript meet PLOS Digital Health’s publication criteria? Is the manuscript technically sound, and do the data support the conclusions? The manuscript must describe methodologically and ethically rigorous research with conclusions that are appropriately drawn based on the data presented.

Reviewer #1: Yes

Reviewer #2: Yes

A. Ok.

--------------------

3. Has the statistical analysis been performed appropriately and rigorously?

Reviewer #1: Yes

Reviewer #2: Yes

A. Ok.

--------------------

4. Have the authors made all data underlying the findings in their manuscript fully available (please refer to the Data Availability Statement at the start of the manuscript PDF file)? requires authors to make all data underlying the findings described in their manuscript fully available without restriction, with rare exception. The data should be provided as part of the manuscript or its supporting information, or deposited to a public repository. For example, in addition to summary statistics, the data points behind means, medians and variance measures should be available. If there are restrictions on publicly sharing data—e.g. participant privacy or use of data from a third party—those must be specified.

Reviewer #1: Yes

Reviewer #2: Yes

A. Ok.

--------------------

5. Is the manuscript presented in an intelligible fashion and written in standard English? PLOS Digital Health does not copyedit accepted manuscripts, so the language in submitted articles must be clear, correct, and unambiguous. Any typographical or grammatical errors should be corrected at revision, so please note any specific errors here.

Reviewer #1: Yes

Reviewer #2: Yes

A. Ok.

--------------------

6. Review Comments to the Author. Please use the space provided to explain your answers to the questions above. You may also include additional comments for the author, including concerns about dual publication, research ethics, or publication ethics. (Please upload your review as an attachment if it exceeds 20,000 characters)

**Reviewer #1:** Thank you for making the recommended changes. This has greatly improved the paper but there are just a couple of minor corrections that are still needed:

•	The incorrect abbreviation FFT is still used in the figure descriptions.

A. So sorry for that. Now, we did a careful searching for the typo, and we hope none still used.

•	Where there are significant effects stated in Table 3, it would be helpful to include p-values (either exact values or something like p < 0.01 or p < 0.001). This could be added to the table or as a footnote with significant values indicated by asterisks in the table.

A. Thanks very much for the suggestions. We added following the recommendations.

There are still a few grammatical errors and I feel that the paper would benefit from some further proof-reading.

A. Thanks again. We made an additional grammatical correction and removed the remaining grammatical errors.

**Reviewer #2:** The writers addressed the comments and provided adequate feedback to the editor and reviewers.

A. Ok.

--------------------

7. PLOS authors have the option to publish the peer review history of their article. If published, this will include your full peer review and any attached files.

Do you want your identity to be public for this peer review? If you choose “no”, your identity will remain anonymous but your review may still be made public.

For information about this choice, including consent withdrawal, please see our Privacy Policy.

**Reviewer #1:** Yes: Sarah Buckingham

**Reviewer #2:** No

A. Thanks for both reviewers for the comments.

--------------------
